# Multiple-Serotype *Salmonella* Outbreaks in Two State Prisons — Arkansas, August 2012

**Published:** 2014-02-28

**Authors:** Rachel E. Gicquelais, Jamae F. Morris, H. Stewart Matthews, Linda Gladden, Haytham Safi, Carla Grayson, Rachel B. Slayton, Anna E. Newton, Rebecca Bordonaro, J. Gary Wheeler, Nathaniel Smith, Stacey A. Bosch, Dirk T. Haselow

**Affiliations:** 1 Arkansas Department of Health; 2 EIS Officer, CDC; 3 Division of Foodborne, Waterborne, and Environmental Diseases, National Center for Emerging and Zoonotic Infectious Diseases, CDC

In August 2012, the Arkansas Department of Health (ADH) was notified of gastrointestinal illness outbreaks in two Arkansas state prisons. ADH investigated the outbreaks and conducted case-control studies to identify the source of the illnesses. This report describes the results of these investigations, which identified 528 persons with onset of diarrhea during August 2–18, 2012. Results from the prison A investigation identified chicken salad as the most likely vehicle. At prison B, person-to-person transmission and contamination of multiple foods likely contributed to illness. Analysis of stool specimens from inmates identified eight serotypes and 15 pulsed-field gel electrophoresis (PFGE) patterns of *Salmonella*. Isolates of *Salmonella* from eggs produced at prison B matched two outbreak patterns. An additional 69 inmates were positive by culture but were not interviewed or did not report diarrhea, making the total case count 597. Sanitarians identified problems with food preparation, hand washing, and food safety training. ADH tested inmate kitchen workers, excluded infected inmates from work, and provided food safety training. Prison kitchen staff should follow guidelines consistent with state regulations for safe food preparation (1) and pass sanitarian inspection.

## Notification of the Outbreaks

On August 6, 2012, ADH learned of an outbreak of diarrhea in approximately 260 inmates at prison A via a local newspaper. ADH began an investigation on August 7. The ADH Public Health Laboratory (ADHPHL) isolated *Salmonella* from stool specimens of seven inmates experiencing diarrhea and identified three serotypes: Anatum, Cerro, and Heidelberg.

On August 14, stool specimens from 16 inmates with diarrheal illness from prison B were sent to a reference laboratory for enteric pathogen testing. On August 21, prison B notified ADH that *Salmonella* was isolated from stool specimens of eight of the 16 inmates. Serotyping completed by ADHPHL on the eight stool isolates identified *Salmonella* Anatum. PFGE patterns were indistinguishable from Anatum isolates from stool specimens of prison A inmates. ADH began a concurrent investigation at prison B on August 22, 8 days after prison B initiated testing.

## Case Finding

Investigators interviewed a convenience sample of 505 (59%) inmates from prison A, 440 (27%) inmates from prison B, and all available staff from both prisons (Table 1). Inmates and prison staff completed questionnaires characterizing food history, symptoms, and symptom onset times. A probable case was defined as self-reported diarrhea with onset during August 2–18, 2012, among prison A or B inmates or staff. A confirmed case was defined as *Salmonella* isolated from a stool specimen during the period of stool specimen testing (August 7–September 25), regardless of the presence or absence of diarrhea. Investigators identified 309 probable and 51 confirmed cases at prison A and 133 probable and 85 confirmed cases at prison B. Of the 360 interviewed persons whose illness met the probable or confirmed case definition at prison A, seven required intravenous rehydration; one experienced acute appendicitis requiring appendectomy, possibly related to the outbreak. No cases from prison B involved complications or receipt of intravenous therapy.

All inmates assigned to kitchen work submitted stool specimens for *Salmonella* testing. Inmates from whom *Salmonella* was isolated were required to submit weekly stool specimens to monitor *Salmonella* clearance. ADHPHL completed serotyping and PFGE on at least one specimen per person by picking a single colony per stool culture plate. Subsequent samples were assessed only for the presence of *Salmonella*. Nineteen additional confirmed cases were identified by stool culture among inmate kitchen workers who were not interviewed.

## Case-Control Studies

Cases were matched to controls by prison housing unit using variable-ratio matching (i.e., the number of controls per case differed for each housing unit). All food items served in the prison cafeterias and commissaries during August 2–5 at prison A and August 7–11 at prison B were included as exposures in conditional logistic regression models. Persons with probable or confirmed illness were excluded from matched odds ratio (mOR) calculations examining food items served after their reported onset date of diarrhea.

At prison A, the 75.1% of persons interviewed who reported consuming chicken salad during lunch on August 4 were much more likely to have probable or confirmed illness than persons who did not report consuming chicken salad (mOR = 7.5; 95% confidence interval [CI] = 4.6–12.7). Given the timing of the chicken salad meal and the peak in cases on August 5 (Figure), a substantial proportion of cases at prison A were likely attributable to consuming the chicken salad. Probable and confirmed cases among persons reporting diarrhea onset before the chicken salad was served also were examined. Fifty cases were identified, two of which were in kitchen workers. One kitchen worker had *Salmonella* Heidelberg (PFGE pattern JF6X01.0022) infection, and the other did not have *Salmonella* isolated from a stool specimen at the time of testing. These persons might have contributed to the early spread of salmonellosis or to contamination of the chicken salad.

At prison B, the 57% of persons interviewed who reported consuming chicken salad for dinner on August 10 were more likely to have probable or confirmed illness than persons who did not report consuming chicken salad (mOR = 4.0; CI = 2.4–6.7). Twenty-three additional food items also were statistically associated with probable or confirmed illness. Inmate interviews did not implicate a single vehicle. One inmate reporting symptom onset on August 2 (Figure) was infected with *Salmonella* Anatum (PFGE pattern JAGX01.0473) and prepared vegetables in the prison B kitchen. Two additional kitchen workers reported symptom onset on August 6. These three persons were not excluded from kitchen work until the ADH investigation began on August 22, 20 days after the earliest reported symptom onset. The prison B outbreak likely was propagated by contamination of multiple foods, although person-to-person transmission also might have perpetuated the outbreak.

## Laboratory Results

ADHPHL cultured stool specimens from 314 inmates; 155 inmates had positive stool cultures for *Salmonella* and were classified as meeting the confirmed case definition. Among the 314 inmates whose stool specimens were cultured, 122 inmates reported diarrhea, and 140 inmates did not report diarrhea. Of the 122 inmates reporting diarrhea, 70.5% tested positive for *Salmonella*. Of the 140 inmates who did not report diarrhea, 35.7% tested positive for *Salmonella*. The remaining 52 inmates tested by stool culture were kitchen workers who were not available for interviews; therefore, symptom information was not obtained. Among the 52 inmate kitchen workers tested and not interviewed, 36.5% tested positive for *Salmonella*.

ADHPHL identified 15 PFGE patterns from *Salmonella* isolated from the 155 positive stool cultures (Table 2). Seven PFGE patterns common to both prisons represented 78% of all stool specimens yielding *Salmonella*; six of these seven patterns had not been isolated previously in Arkansas. The seventh pattern, *Salmonella* Adelaide (PFGE pattern TDAX01.003AR), was isolated only once previously, in 2008, from a child whose father worked at prison B. Weekly stool specimens were submitted by 137 inmate kitchen workers to ensure *Salmonella* clearance. Among 31 persons who had multiple specimens serotyped, 10 had two or more serotypes identified (Table 2).

## Environmental Investigations

ADH sanitarians inspected each prison’s kitchen and dining facilities after receiving reports of illness. Sanitarians documented multiple violations of the Arkansas State Board of Health’s *Rules and Regulations Pertaining to Food Establishments* (1). During four inspections conducted by ADH sanitarians at prison A on August 6–15, violations included neglect of hand washing among inmates; inadequate freezing, cooling, and reheating procedures; moldy ceilings; unclean equipment and surfaces; and cracked, noncleanable food storage containers, food preparation surfaces, walls, and floors. Hand washing sinks required hand contact to operate and were below standard height.*

Interviews with prison A kitchen workers were conducted to characterize the preparation of the chicken salad served for lunch on August 4. Along with video surveillance footage, interviews revealed that the cooked chicken was not refrigerated and was held at an ambient temperature of approximately 75°F–99°F (23.9°C–37.2°C) for 15 hours before incorporation into the chicken salad. Inmates were unsupervised during much of the meal preparation.

Violations documented during an August 28 inspection of the prison B kitchen included absent temperature monitoring during cooking and noncleanable, cracked floors and food storage containers. Rodents and cockroaches infested both facilities. Neither facility provided food safety training to kitchen workers. Additionally, neither facility required ill workers to report symptoms to management, nor did they ensure ill workers were restricted or excluded from working with food. Both facilities passed ADH sanitarian inspection <6 months before the outbreaks; however, review of the inspection records revealed that the inspections did not fully adhere to ADH inspection guidelines for commercial food establishments. In Arkansas, prisons are required to follow the same regulations as commercial food establishments and are subject to periodic inspection by ADH sanitarians.

Because prison B supplied itself and other state prisons, including prison A, with eggs from its three hen houses during August 2012, ADH sanitarians inspected the prison B hen houses and egg processing procedures and equipment. Prison B officials revealed that their outdoor egg washer required frequent maintenance and was replaced with an indoor washer in August 2012. Both prisons incorporated eggs produced at prison B into the chicken salad dishes served on August 4 and 10 at prisons A and B, respectively.

## Food Item Testing

On January 24, 2013, 12 raw, nonsanitized eggs were collected from one of the prison B hen houses. The two other hen houses that were operational during August 2012 were demolished during September–December 2012. ADHPHL cultured each egg sample using four types of selective media and selected two colonies with suspected *Salmonella* morphologies from each culture plate for biochemical testing. Of the 96 candidate colonies subject to biochemical testing, 17 were identified as *Salmonella*. PFGE patterns of the 17 egg isolates were indistinguishable from *Salmonella* Adelaide and *Salmonella* Cerro patterns from nine stool specimens from inmates at both prisons (Table 2).

Several other food items were collected for *Salmonella* testing during August 7–September 13, 2012. Samples of several meals were collected on August 7 from prison A and on August 22 from prison B, including the chicken salad served on August 4 at prison A, frozen samples of the chicken salad served on August 10 at prison B, and frozen samples of the meatloaf and baked chicken served for lunch and dinner, respectively, on August 11 at prison B. Additionally, several food items not consumed by inmates or prison staff during the outbreak period but representative of ingredients used in meals served during August 2012 were collected. These included raw, frozen chicken collected from prison A on August 24, raw, frozen chicken collected from prison B on August 22, and salad dressing used in the chicken salad recipes at both prisons from the Arkansas correctional system’s food supplier warehouse on September 13. All items tested negative for *Salmonella*, with the exception of raw, frozen chicken from prison B, which tested positive for *Salmonella* Enteritidis, a *Salmonella* serotype not identified in stool specimens from inmates at either prison.

## Public Health Response

All inmate kitchen workers were required to submit stool specimens for testing. Inmates testing positive for *Salmonella* submitted weekly stool specimens for testing and were excluded from kitchen work until two successive stool specimens were negative for *Salmonella*, *Shigella*, *Escherichia coli*, and *Campylobacter* and diarrheal symptoms resolved. Exclusion of inmate kitchen workers at prison B was delayed because of a 20-day lapse from the earliest reported symptom onset date to the beginning of the ADH investigation. ADH sanitarians provided recommendations and food safety training, emphasizing compliance with published guidelines (1). Inmate transfers and releases were suspended until the outbreaks were controlled. Ciprofloxacin treatment was recommended for patients at risk for systemic disease, in accordance with published guidelines (2).

### Editorial Note

This report describes two large, multiple-serotype *Salmonella* outbreaks associated with food preparation deficiencies. Inadequately sanitized eggs provided to both prisons were a potential source for at least two of the *Salmonella* PFGE patterns involved. Among the sample of 1,047 inmates and prison staff interviewed, 64.1% and 44.9% at prisons A and B, respectively, had illness that met the probable case definition (i.e., reported diarrhea) or met the confirmed case definition after having *Salmonella* isolated by stool culture, with or without reporting symptoms of diarrhea. Additional cases likely existed among noninterviewed and untested inmates beyond the 597 total cases identified in the investigation.

What is already known on this topic?

*Salmonella* is the most common cause of bacterial foodborne illness the United States; however, multiple-serotype *Salmonella* infections and outbreaks are identified infrequently.

What is added by this report?

Two linked *Salmonella* outbreaks occurred in Arkansas prisons during August 2012, revealing 15 pulsed-field gel electrophoresis patterns of *Salmonella* and 10 inmates with multiple-serotype infections. Deficiencies in safe food preparation practices, a lack of inmate kitchen worker training, neglect of hand washing, a delay in recognition and reporting of one of the outbreaks, and inadequately sanitized eggs produced by and distributed to the prisons might have influenced the occurrence, size, and transmission modes associated with the outbreaks.

What are the implications for public health practice?

Correctional facility and inmate food service personnel should receive food safety training. Prisons should be inspected by sanitarians in accordance with state or local guidelines and should maintain equivalent standards to commercial food service establishments.

Multiple-serotype outbreaks of *Salmonella* have been reported in prisons previously (3); however, the number of serotypes in these outbreaks surpasses all previous reports. These outbreaks demonstrated different epidemiologic characteristics, one primarily involving point-source contamination of chicken salad, and the other potentially involving multiple transmission modes and vehicles. These outbreaks show that environmental and food preparation practices can affect the course and extent of an outbreak caused by the same pathogen.

Ten cases of infection with multiple serotypes of *Salmonella* were identified. Multiple-serotype infection in individuals is reported infrequently (4). Additional multiple-serotype infections in these outbreaks likely were missed because laboratory testing of follow-up samples was limited to ascertaining whether *Salmonella* was present. Further, only one stool culture medium was used, although detection of specific serotypes is influenced by enrichment medium choice (5). The detection of multiple serotypes in different stool specimens over time might indicate coinfection. Persons infected with multiple serotypes also could clear one serotype before another, manifesting differential serotype survival. Furthermore, the persistence in the gut or infectious periods of *Salmonella* serotypes might differ. These limited data indicated that among persons with multiple-serotype infections, serotype Anatum was present in 90% of cases; however, no clear progression was observed from infection with one serotype to infection with a second. The effect of multiple-serotype infection on *Salmonella* shedding and pathogenesis is unknown.

Asymptomatic carriage was identified in 50 confirmed cases; 56% were infected with *Salmonella* Anatum pattern JAGX01.0473. The combination of 15 serotypes, 10 multiple-serotype infections, and asymptomatic infection among 32.3% of confirmed cases might illustrate the persistence of certain *Salmonella* serotypes among the prison population. Because *Salmonella* colonization among poultry has been demonstrated (6) and two of the 15 outbreak serotypes were isolated from nonsanitized eggs collected from a prison B hen house, the outbreak strains might colonize laying hens from prison B. Laboratory testing of nonfood items, including laying hens, was outside of the scope of this investigation. Although it was not possible to describe the *Salmonella* serotypes colonizing poultry from prison B beyond their identification in eggs, the propensity of *Salmonella* to colonize poultry further highlights the need for safe cooking and food storage practices to kill *Salmonella* and prevent its growth in contaminated food before consumption.

Prisons should follow safe food preparation guidelines (1). Inmates should receive food safety training before assignment to kitchen work. Sanitarians should regularly inspect prison kitchens, cafeterias, and agricultural facilities, and require them to maintain standards equivalent to those of commercial establishments in accordance with state or local guidelines. Health departments might consider enhancing collaborative surveillance with prison staff to improve control of foodborne outbreaks in prisons.

## Figures and Tables

**FIGURE:**
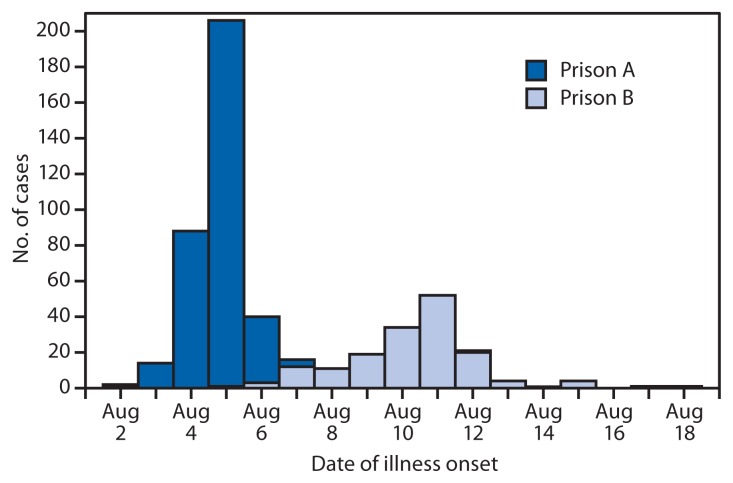
Number of confirmed and probable salmonellosis cases at prisons A and B^*^ — Arkansas, August 2012 ^*^ N = 514 cases (350 at prison A and 164 at prison B) with a reported symptom onset date.

**TABLE 1:** Interviews and laboratory testing among prison A and B inmates and staff — Arkansas, August 2012

Prison	Prison subgroup	No. interviewed	Reported diarrhea		No. tested*	Laboratory confirmed†	
	
			No.	(%)		No.	(%)
A§	Staff	57	15	(26.3)	0	0	—
	Inmate kitchen workers	68	48	(70.6)	89	52	(58.4)
	Inmates not assigned to kitchen	437	288	(65.9)	7	4	(57.1)
	**Total**	**562**	**351**	**(62.5)**	**96**	**56**	**(58.3)**
B¶	Staff	45	3	(6.7)	0	0	—
	Inmate kitchen workers	190	58	(30.5)	194	85	(43.8)
	Inmates not assigned to kitchen	250	116	(46.4)	24	14	(58.3)
	**Total**	**485**	**177**	**(36.5)**	**218**	**99**	**(45.4)**

* Stool specimens were tested for *Salmonella* using standard microbiologic techniques. Serotyping and pulsed-field gel electrophoresis were completed for at least one sample per person.

† 19 confirmed cases were excluded from the case-control analyses because the case-patient was not interviewed.

§ Prison A housed 849 inmates during August 2012.

¶ Prison B housed 1,616 inmates during August 2012.

**TABLE 2:** Serotypes and pulsed-field gel electrophoresis (PFGE) patterns of *Salmonella* isolates from positive stool cultures at two prisons, and from prison B eggs — Arkansas, August 2012

Serotype	PFGE pattern	No. of isolates at prison A	No. of isolates at prison B	No. of isolates in prison B eggs
Adelaide	TDAX01.003AR	1	5	9
Anatum	JAGX01.0474	5	7	0
Anatum	JAGX01.0473	5	73	0
Anatum	NA*	0	2	0
Braenderup	JBPX01.0007	12	5	0
Cerro	JCGX01.0060	6	5	0
Cerro	JCGX01.003AR	1	2	8
Cerro	JCGX01.004AR	0	1	0
Cerro	JCGX01.005AR	0	3	0
Cerro	JCGX01.006AR	0	1	0
Heidelberg	JF6X01.0022	20	0	0
Heidelberg	JF6X01.0052	2	0	0
Litchfield	JGXX01.0010	0	1	0
Mbandaka	TDRX01.0373	2	1	0
Newport	JJPX01.0056	5	0	0
Newport	JJPX01.4010	1	0	0
**Total**	**15**	**60** †	**106** §	**17**

* Not available (PFGE analysis not completed).

† A total of 60 *Salmonella* isolates were cultured from 56 patients; three patients had multiple-serotype infections. Two patients were infected with two serotypes of *Salmonella*. One was infected with Cerro and Newport, and the second was infected with Anatum and Heidelberg. One patient was infected with three serotypes of *Salmonella* (Anatum, Cerro, and Heidelberg).

§ A total of 106 *Salmonella* isolates were cultured from 99 patients; seven patients had multiple-serotype infections. Six patients were infected with serotypes Anatum and Cerro. One patient was infected with serotypes Anatum and Braenderup.

## Footnotes

* The height of hand washing sinks at prison A was approximately 24 inches (62 cm). ADH recommended installing sinks at a height of approximately 36 inches (91 cm).
